# Achieving enhanced stabilization and controlled release of curcumin *via* cross-linked polydopamine particles

**DOI:** 10.1016/j.heliyon.2024.e41379

**Published:** 2024-12-20

**Authors:** Majid Moussaei, Ebrahim Tajik, Vahid Haddadi-Asl, S. Ali Mazloumi, Helia Heydarinasab, Elahe Abdollahi, Fatemeh Haj-Sadeghi, Hanie Ahmadi, Mohammad Reza Gholizadeh

**Affiliations:** Department of Polymer Engineering and Color Technology, Amirkabir University of Technology, P.O. Box 15875-4413, Tehran, Iran

**Keywords:** Polydopamine, Curcumin, Controlled drug release, Molecular simulation

## Abstract

Development of efficient drug delivery systems remains a critical challenge in pharmaceutical applications, necessitating novel approaches to improve drug loading and release profiles. In this study, a novel method is presented for fabricating crosslinked polydopamine particles (XPDPs) using a water/water Pickering emulsion system. The emulsion is composed of poly(ethylene glycol) and dextran, stabilized by polydopamine (PDA) particles. This method yields XPDPs with a mean particle size of 0.55 μm, significantly smaller than PDA particles (1.025 μm), resulting in a higher surface area favorable for drug loading. The adsorption mechanism involves electron sharing and covalent bonding between the carrier and drug molecules. The adsorption, release, and drug delivery kinetics of the XPDPs are compared with those of the non-crosslinked PDA particles. The results demonstrate that XPDPs exhibit improved adsorption properties due to their crosslinked structure and increased positive charge due to presence of secondary amines. During a 28-h period, curcumin release from PDA declines from around 80 %–40 %, while for XPDA, it decreases from approximately 60 %–35 % as the pH shifts from 7.4 to 5. While PDA particles display a burst release profile, XPDPs show a more gradual and sustained release, attributed to their enhanced structural stability. Molecular simulations were conducted to estimate the solubility parameters, confirming the compatibility between PDA and dextran for effective drug loading.

## Graphical abstract

**Figure:**
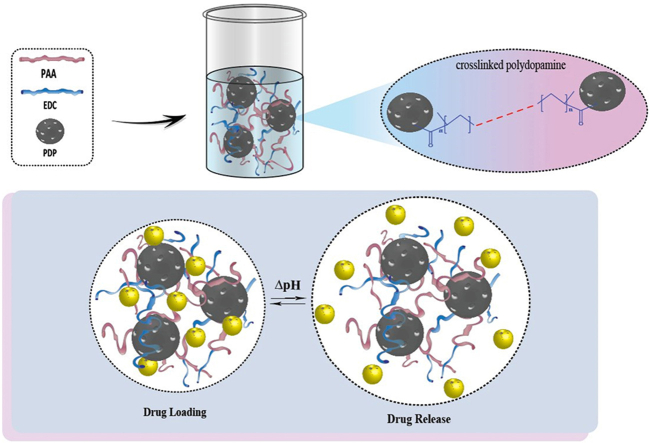

## 1 Introduction

Polydopamine (PDA) has gained significant attention in the biomedical field due to its unique properties, including biocompatibility, non-toxicity, and excellent adhesive capabilities, derived from its mussel-inspired structure and functional groups, originated from PDA's melanin-mimicking structure and the catechol groups inherited from its dopamine monomer [[1], [2], [3], [4], [5], [6], [7], [8]]. The facile synthesis of PDA in alkaline solutions has led to its widespread application in tissue engineering, medical implants, and drug delivery systems [[9], [10], [11]]. Recent advancements demonstrated capacity of PDA-based nanoparticles for photothermal conversion efficiency, rendering them a great candidate for photothermal and chemodynamic therapies. PDA's functional groups facilitate the secondary modifications that enhance its versatility for drug delivery and other therapeutic applications [7,12,13].

PDA particles in different shapes like mesoporous [11], walnut-shaped [4], hollow [14], nanospheres [15], and nano bottles [16] have become more attractive for drug delivery of various drugs. Microspheres or microparticles offer many advantages such as controlled drug release, different administration routes, targeted drug delivery, drug safety, and fewer side effects [17]. Therefore, the loaded drug can be suspended, dispersed, and dissolved in the microsphere matrix or adsorbed on its surface [18]. In addition, surface-activated polydopamine microspheres can absorb drugs through interactions [[19], [20], [21]]. In the research by Liu et al. [4], the drug release exhibited a consistent and linear pattern, attributed to the numerous π-π interactions facilitated by the aromatic rings in the anticancer drug, doxorubicin. However, the desired drug release period did not align with mesoporous particles. Wang et al. [22] effectively highlighted the potential of mesoporous particles in their research on cartilage and joint regeneration.

As previously mentioned, PDA serves as a suitable carrier for achieving desired release over various time intervals, ranging from hours to days, and it can be adjusted by modifying key parameters [4,5,22]. Pickering emulsions refer to some new emulsions stabilized with solid particles instead of conventional surfactants [[23], [24], [25], [26], [27]]. The particles form a solid, dense multi or single film around the emulsion droplets through interfacial interactions to protect them from aggregation. This mechanism is currently defined by mechanical barrier theory; by this approach, different kinds of emulsions could be prepared. Emulsions are widely used to protect drugs in drug delivery applications. As water-based polymer systems are increasingly popular due to less contamination, water-in-water (W/W) emulsions are widely considered [24,25,28,29]. Nishizawa et al. [26] used PDA particles to stabilize W/W and oil-in-water emulsions *via* crosslinking the particles. It must be noted that W/W emulsions cannot be stabilized with surfactants.

**Fig. 1:**
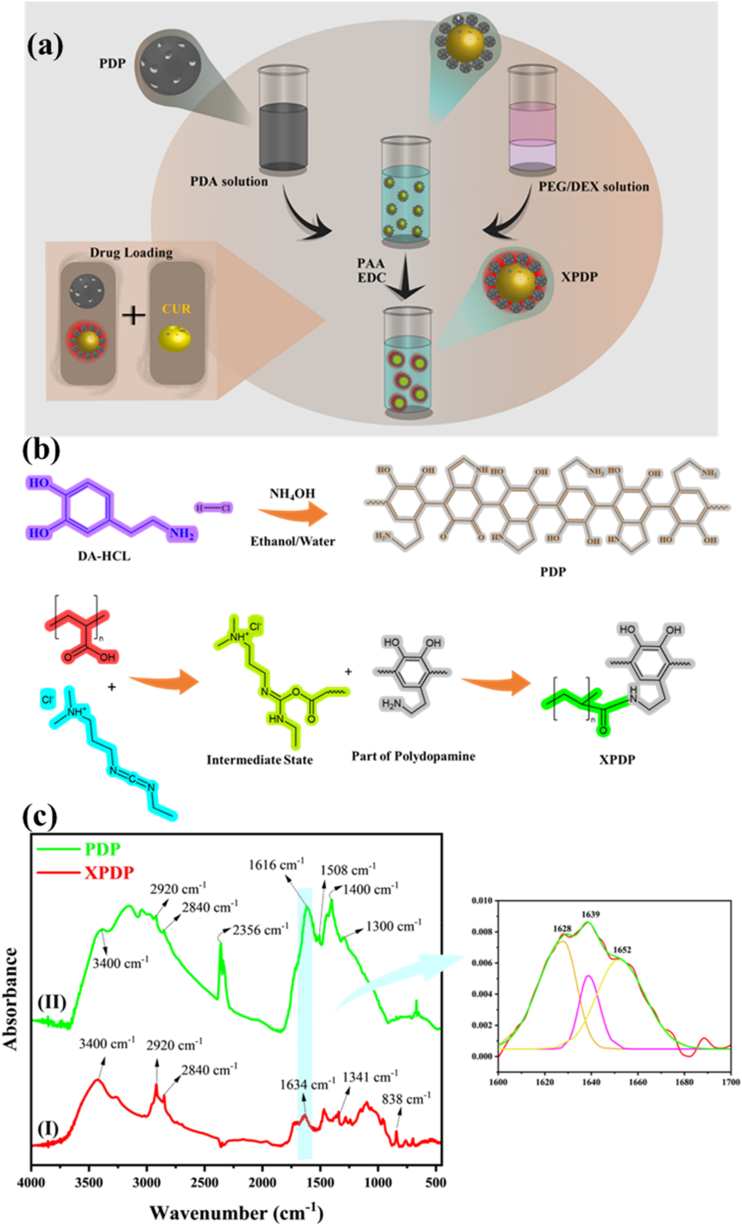
(a) Schematic representation of XPDPs synthesis. (b) Synthesis procedure of PDP and XDPD. (c) FTIR spectra for I) XPDP and II) PDA, along with the deconvoluted amide region for XPDP.

In this study, PDA microparticles are initially synthesized. Subsequently, a common aqueous emulsion or aqueous biphasic system is created using dextran and poly(ethylene glycol) (PEG), which is then stabilized through the cross-linking of synthetic PDA microparticles (XPDP). In the synthesis of the crosslinked PDA particles (XPDPs), *N*-ethyl-*N*′-(3-dimethylaminopropyl) carbodiimide hydrochloride (EDC) and poly(acrylic acid) (PAA) are utilized. EDC is a common coupling agent that facilitates the formation of amide bonds between the amine groups of PDA and the carboxyl groups of PAA. This crosslinking reaction helps to stabilize the PDA structure and create a more rigid particle matrix. The inclusion of PAA also introduces additional carboxyl groups onto the particle surface, which can further enhance drug loading capacity through electrostatic interactions and hydrogen bonding with drug molecules. The use of EDC and PAA crosslinking is a key step in the fabrication of the XPDPs, as it imparts improved structural integrity and versatility for drug delivery applications compared to the non-crosslinked PDA particles This method was chosen to create a stable XPDP structure with potentially higher surface area for efficient drug loading, such as the model drug curcumin. The drug loading efficiency and release behavior of curcumin are investigated on both PDA microparticles and XPDPs. The performance of XPDPs is compared with that of PDA microparticles to elucidate the impact of crosslinking on drug adsorption and release kinetics. Additionally, the influence of environmental pH on the drug release profile is explored to gain insights into the potential for pH-responsive drug delivery. Molecular simulations are employed to estimate solubility parameters, providing a theoretical foundation for the compatibility between PDA and dextran in the system (Fig. 1(a)).

## 2 Materials and methods

### 2.1 Materials

2-(3,4-Dihydroxyphenyl)ethylamine hydrochloride (dopamine hydrochloride, DA-HCl), ethanol (99 %), ammonium hydroxide (NH_4_OH; 25 %), dextran (analytical grade, Mw = 400 g mol^−1^), poly(ethylene glycol) (PEG; Mw = 35000 g mol^−1^, analytical grade), *N*-ethyl-*N*′-(3-dimethylaminopropyl) carbodiimide hydrochloride (EDC), and poly(acrylic acid) (PAA, Mw = 450000 g mol^−1^) were purchased from Sigma-Aldrich.

### 2.2 Synthesis of polydopamine

A typical mixed-solvent method was used for the synthesis of PDA. The amount of DI water, ethanol, NH_4_OH, and DA-HCl were 50 mL (2.778 mol), 20 mL (0.343 mol), 0.375 mL (0.009 mol), and 0.25 g (0.002 mol), respectively. A two-necked flask containing ethanol, NH_4_OH, and 45 mL DI water was stirred for 30 min at 300 rpm while it was sealed and the temperature was kept at 30 ± 2 °C. Meanwhile, DA-HCl was mixed with the rest of the water and injected into the medium, and the reaction was allowed for 30 h. After dopamine addition, the solution's brown-creamy color was vivid. When the reaction was completed, the pure black solution was centrifuged at 5000 rpm and washed with water three times. The obtained PDA was dried in the oven at 80 °C for 24 h. The reaction conversion was obtained 0.72.

### 2.3 Water-in-Water emulsion and crosslinking

As shown in Figs. 1(a), 900 mg dextran and 400 mg PEG were dissolved in DI water individually. These two solutions were mixed (70:30 wt/wt.) while adding 3 mL PDA suspension (0.2 g L^−1^). The mixture was stirred *via* Vortex for 1 min at 3500 rpm. Dextran/PEG emulsions have been prepared but not stabilized without the presence of dopamine cross-linked particles. The pre-prepared PAA (100 mM) and EDC (120 mM) aqueous solutions were added to the mixture in precise amounts. 0.5 mL the former solution was added to the mixture, and 0.4 mL the latter was dropped in. Afterward, the media was stirred *via* Vortex at 3500 rpm for 3 min. Then, the so-called W/W emulsion stabilized with XPDPs throughout this process. The whole mixture was poured into a Petri dish and dried for 24 h in the oven at 80 °C. The obtained powder was washed with distilled water several times and dried at 50 °C.

### 2.4 Characterization methods

The Thermo Fish spectrometer was used to obtain Fourier-transform infrared (FTIR) spectra, capturing 20 scans for each sample at a resolution of 4 cm^−1^. The samples were prepared by mixing 10 mg of powdered solid sample with 100 mg of potassium bromide (KBr) and compressing the powder mixture into tablets under a pressure ranging from 10,000 to 15,000 psi. The analysis was conducted within the 400-4000 cm^−1^ range. Subsequently, scanning electron microscopy (SEM) was performed using the AIS2100 microscope from SERON Technologies, operating at 25 kV, to visualize the samples' morphology. The process of preparing the sample involved transforming the powdered solid sample into a tablet. Using a UV/Visible spectrophotometer (Rayleigh-UV2601 UV/VIS, China), the loading efficiency was calculated. For this purpose, 5 mg of fully dried nanoparticles were dissolved in 10 mL phosphate-buffer saline (PBS). The zeta potential was performed using SZ-100z, Horiba Jobin Yvon company zeta potentiometer. The X-ray diffraction (XRD) curve performed using an X-ray diffractometer (PHILIPS PW1730). The BET analysis was applied to measure the surface area.

### 2.5 Solubility parameter

The solubility parameter was estimated using Materials Studio software with the assistance of coherent energy density. The mentioned parameters for dopamine and curcumin were calculated after the preparation of the molecule using the Forcite dynamic module. The aforementioned calculations were carried out for PDA with 18 repeating units and dextran with 3 repeating units.

### 2.6 Drug loading and release studies

#### 2.6.1 Examining adsorption and release properties

To examine the impact of the initial pH of solution on the adsorption characteristics of the anionic curcumin drug, adsorption tests were conducted on both PDA and XPDA carriers. The tests involved exposing a drug solution with an initial concentration of 100 ppm to 1 mg of adsorbent in a 10-mL vial, maintained at 298 K for 24 h. Subsequently, the equilibrium adsorption capacity was determined using Equation (1) [30,31]:

(1) $$q_{e}=\frac{\left(C_{0}-C_{e}\right)V}{m}$$

In the aforementioned equation, the parameter *q*_*e*_ (mg.g^−1^) represents the equilibrium adsorption capacity. *C*_*0*_ (ppm) and *C*_*e*_ (ppm) correspond to the concentration of the drug in the initial solution and the equilibrium solution after the 24-h period, respectively. *V* (L) denotes the volume of the solution, while *m* (g) represents the mass of the absorber.

#### 2.6.2 Investigation of adsorption kinetics

To study the adsorption kinetics, 1 mg XPDA was mixed with 10 mL drug solution with an initial concentration of 20 ppm at pH = 2 and 298 K for 24 h. The drug concentration was then measured using a UV–Vis spectrophotometer at specific time intervals after centrifuge sedimentation. The adsorption kinetics of curcumin were also investigated under constant temperature, pH, and initial adsorbent concentration. The adsorption capacity was measured at different time points, and the adsorption capacity at the desired time was calculated using Equation (2):

(2) $$q_{t}=\frac{\left(C_{0}-C_{t}\right)V}{m}$$

Regarding this matter, the adsorption capacity at time *t* is represented by *q*_*t*_ (mg.g^−1^), while the concentration of curcumin in the solution is denoted as *C*_*t*_ (ppm). Two linear models, namely the pseudo-first-order and pseudo-second-order models, were employed for the investigation. The models involved calculating the slope and intercepting from the origin of the (ln*(q*_*e*_*-q*_*t*_*)*) vs. (*t*) and (*t/q*_*t*_) vs. (*t*)graphs, allowing for the determination of the equilibrium adsorption capacity (*q*_*e*_) and the constants *k*_*1*_ and *k*_*2*_. The *q*_*e*_ and *q*_*t*_ respectively refer to the equilibrium adsorption capacity and the adsorption capacity at time *t* (in minutes) measured in mg.g^−1^. The constants *k*_*1*_ and *k*_*2*_ represent the pseudo-first-order and pseudo-second-order constants, respectively, with units of min^−1^ and g.min^−1^.mg^−1^as shown in Equation (3) and Equation (4) [32,33].

(3) $$\ln\left(q_{e}-q_{t}\right)=\ln\left(q_{e}\right)-k_{1}t$$

(4) $$t/q_{t}=1/k_{2}{q_{e}}^{2}+t/q_{e}$$

In order to investigate the intraparticle penetration process, Equation (5) (Weber equation) has been used:

(5) $$q_{t}={k_{i}t}^{0.5}+c$$

The rate constant *k*_*i*_ (mg g^−1^ min^0.5^) and the parameter *c* (mg.g^−1^) in the given equation can be determined by analyzing the slope and intercept of the linear graphs involving *q*_*t*_ as a function of *t* ^*0.5*^. Additionally, Boyd's kinetic model was employed to differentiate between surface layer penetration and internal particle penetration during the adsorption process. According to this model, it is assumed that the primary resistance to adsorption is concentrated within the adsorbent boundary layer. The model is expressed in Equation (6):

(6) $$\ln\left(1-F\right)=-k_{f}t$$

In the equation provided*, k*_*f*_ (min^−1^) represents the infiltration rate constant in the surface layer, and *F* is equilibrium fraction at various time points, which can be determined by Equation (7):

(7) $$F=\frac{q_{t}}{q_{e}}$$

#### 2.6.3 Adsorption isotherms

Adsorbent with a 10 ml curcumin solution containing 20–100 ppm drug sonicated at pH ⁓ 2. Following this, the mixture was agitated using a magnetic stirrer at 150 rpm in a water bath maintained at 298 K for 8 h to achieve equilibrium. *C*_*e*_ (ppm) represents the equilibrium concentration of the drug, *qe* (mg.g^−1^) denotes the adsorption capacity at equilibrium, and $q_{0}$ (mg.g^−1^) signifies the maximum adsorption capacity. The Langmuir constant *K*_*L*_ (L.mg^−1^), the Freundlich constant *K*_*f*_ (g^−1^ mg^1−(1/n)^ L^1/n^), and the parameter *n* are associated with the adsorption intensity in the Freundlich model as illustrated in Equations (8), (9) [34,35].

(8) $$C_{e}/q_{e}=1/q_{0}k_{L}+1/q_{0}C_{e}$$

(9) $$\ln\;q_{e}=\ln\;k_{F}+1/n\;\ln\;C_{e}$$

#### 2.6.4 Investigation of curcumin release properties

To assess the release of curcumin, it was loaded on XPDA and PDA. After 24 h, the remaining drug concentration in the precipitated solution was measured using a centrifuge and a spectrophotometer, and the adsorption capacity was calculated. Following the drying of the adsorbent in a vacuum oven at 40 °C for 12 h, 1 mg of the loaded adsorbent was dispersed in 10 mL of buffer solution with varying pH levels, and the reaction vessels were placed in a water bath at 30 °C. At specific time intervals, the suspensions were centrifuged, and 3 mL of the upper solutions were removed. To create a concentration gradient, 3 mL of fresh buffer was added to the suspensions. The samples were analyzed by a spectrophotometer to determine the concentration of the released drug, and the cumulative concentration at any time was calculated using the following equation:

(10) $$C_{n}^{\prime }=\frac{C_{n}V_{T}+\sum_{i=1}^{i=n-1}C_{n,i}V_{S}}{V_{T}}$$

In the above relation *C*^*'*^_*n*_ (ppm) is cumulative concentration at time *n*, *C*_*n*_ (ppm) is concentration at time *n*, *C*_*n-1*_ (ppm) is concentration at time *n-1*, *V*_*t*_ (L) is total solution volume, and *V*_*s*_ (L) is volume of removed sample.

## 3 Results and discussion

### 3.1 Characterizations

As mentioned in previous sections, the synthesis of PDA is carried out with the assistance of ammonium chloride in a water-alcohol solvent. The structure of PDA has been discussed for many years, and a specific and precise illustration is still lacking. The structure depicted in Fig. 1(b) has been drawn based on previous studies of this polymer, showing a hexamer [11]. Additionally, multiple functional groups can be observed in its cyclic structure, including hydroxyl, amine, and carbonyl groups. It is worth noting that the carbonyl groups have emerged due to the rearrangement of π bonds. In the crosslinking section (Fig. 1(b)), there are carboxylic acid groups available as a side group of the polymer and EDC as the crosslinker. The carbonyl oxygen attacks the carbodiimide group and opens the double bond, resulting in an ester formation of O-acylisourea that easily and quickly reacts with primary amine *via* nucleophilic attack. This reaction will form an amide group with carboxylic acid, and the EDC urea-derivative by-product will be soluble in the media. As primary amine groups are present in polydopamine, covalent bonds can be formed between different chains of polymers and PAA, resulting in crosslinked polydopamine [36].

Fourier-transform infrared (FTIR) spectra of PDA and XPDP particles reveal critical insights into their structural differences and confirm successful crosslinking in XPDP (Fig. 1(c)). Both spectra exhibit a broad peak around 3400 cm⁻^1^, corresponding to O-H and N-H stretching vibrations associated with the hydroxyl groups of catechol and amines in the polydopamine structure. PDA also shows characteristic peaks at 2920 cm⁻^1^ and 2840 cm⁻^1^ for aliphatic C-H stretching, and at 1600 cm⁻^1^ and 1500–1508 cm⁻^1^ for C=C aromatic ring stretching and N-H bending vibrations. The XPDP spectrum reveals distinct modifications, most notably a new peak around 1630–1634 cm⁻^1^, which indicates the formation of secondary amide bonds from the reaction between the amine groups of PDA and the carboxyl groups of the crosslinking agent, such as polyacrylic acid (PAA). Additional features in XPDP include peaks at 1341 cm⁻^1^, likely from C-N stretching due to crosslinking, and the appearance of an 838 cm⁻^1^ peak associated with new C-C bonds. Peaks in the 2800–2900 cm⁻^1^ range suggest the formation of amine salts through protonation of amine groups, enhancing particle stability and drug-loading capacity. Detailed analysis of the 1600–1700 cm⁻^1^ region shows peak deconvolution at 1628, 1639, and 1652 cm⁻^1^, corresponding to various C=C and C=O vibrations, further confirming structural modifications [[37], [38], [39]]. These spectral changes highlight the successful crosslinking of PDA to produce XPDP, resulting in enhanced structural integrity and properties suitable for biomedical applications, including improved drug loading and controlled release. In order to investigate structure more precisely, the XRD analysis was preformed, and the result is presented in Fig. S1.

**Fig. 2:**
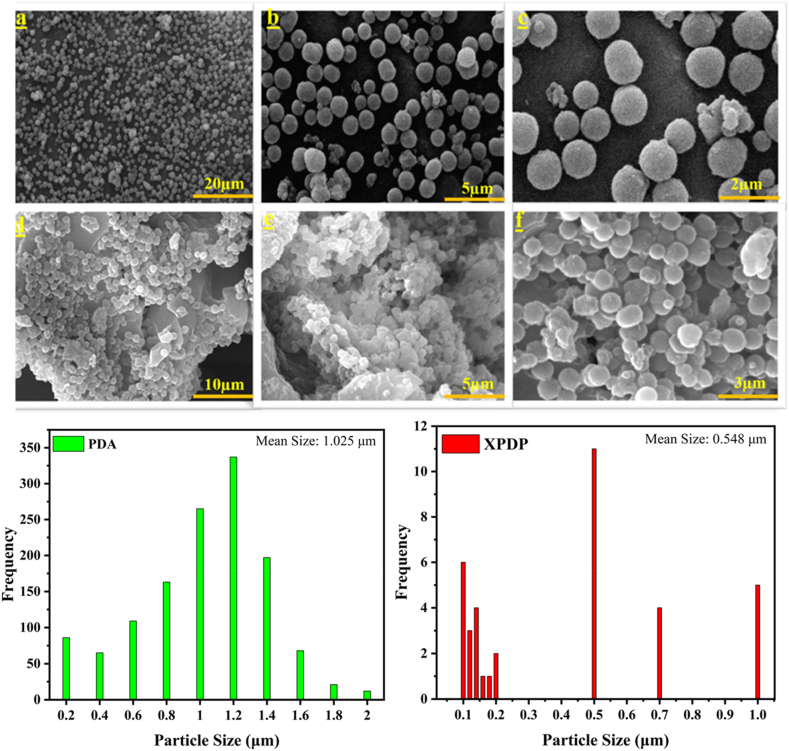
The SEM images of (a–c) polydopamine particles (PDA) and (d–f) crosslinked polydopamine (XPDP), along with the particle size distribution for each sample.

As shown in Fig. 2(a–c), the SEM images confirm the successful synthesis of PDA microparticles *via* the described process. The spherical morphology of the PDA particles is evident, with minimal aggregation observed, as reflected in the well-dispersed arrangement of the particles. The particles exhibit a smooth surface and consistent size distribution, highlighting the precision of the synthesis method. In contrast, Fig. 2(d–f) depicts the morphology of the crosslinked XPDP particles. While the spherical shape is generally retained, the surface of these particles appears rougher compared to the PDA particles, which may be indicative of the crosslinking process. Additionally, the size of the XPDP particles is notably smaller than that of the PDA particles, with the particle size distributions shown in the histograms (Fig. 2, bottom panels). The mean particle size for PDA is 1.02 μm, while for XPDP, it is significantly reduced to 0.55 μm, illustrating the effect of the crosslinking process on particle dimensions. The reduction in particle size and the increase in surface roughness result in a higher surface area for XPDP particles. This enhanced surface area can make the XPDP particles particularly suitable for applications requiring higher loading capacities, such as drug delivery systems, where increased drug adsorption and controlled release properties are desired [[40], [41], [42], [43]]. For measuring surface area, BET analysis was done. The results showed that the surface area of XPDP is about 48 m^2^/g.

### 3.2 Investigating the effect of pH on adsorption properties

**Fig. 3:**
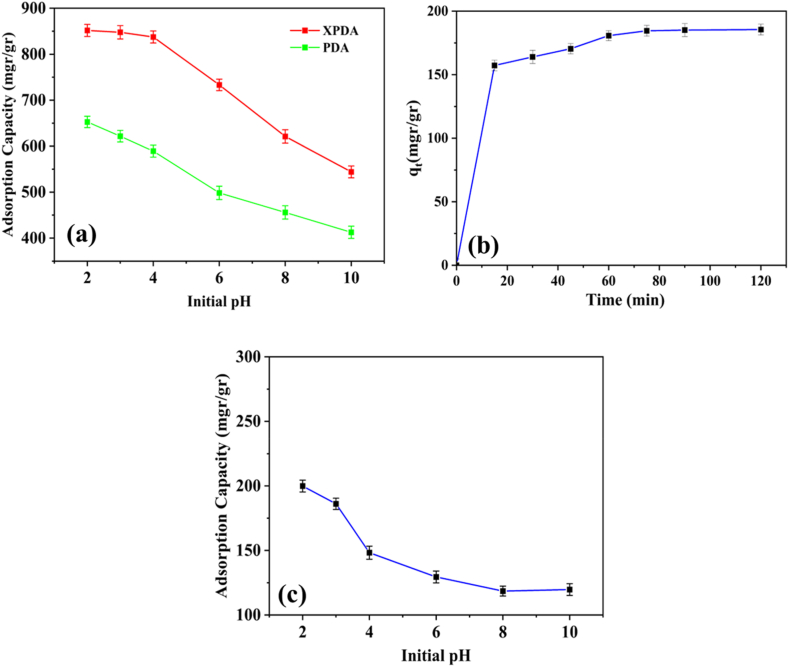
(a) The adsorption capacity of PDA and XPDA at different pH values (b) XPDA adsorption capacity in terms of time at the initial concentration of 100 ppm curcumin and pH⁓2 (c) XPDA adsorption capacity at different pH values in the initial concentration of 100 ppm curcumin and pH ⁓2.

The pH of the medium significantly influences the adsorption and release properties. Dopamine's isoelectric point is around 3.5, resulting in a negative charge at high pH and a positive charge at lower pH. As the pH values increase from 2 to 10, the adsorption capacity decreases from 652.70 mg g^−1^ to 412.70 mg g^−1^ for PDA and from 851.60 mg g^−1^ to 544.11 mg g^−1^ for XPDA. These findings suggest that at low pH, the electrostatic attraction between the anionic drug curcumin and the polydopamine coating increases the equilibrium adsorption capacity, as the concentration of H^+^ rises below the isoelectric point, leading to the protonation of amine groups (-NH_2_- and -NH-) on the absorbent surface as -NH^3+^- and -NH^2+^-. Additionally, the equilibrium adsorption capacity in XPDA is higher than in PDA, possibly due to the cross-links strengthening the outer surface of the carrier and the system's stability, as well as the presence of a second type of amine in the structure, which increases the positive charge. Furthermore, the presence of aromatic rings in the structure of polydopamine and curcumin molecules contributes to the high adsorption capacity, likely due to the π-π interactions between the carrier and the drug. These results underscore the significant role of pH and electrostatic forces in the adsorption process, with higher adsorption capacity at high pH and improved adsorption properties due to the presence of cross-links. Fig. 3(a) shows the adsorption capacity of PDA and PDA at different pH values. The zeta potential values were measured at various pH and the results are presented in Supplementary Material (Fig. S2). The low values of zeta potential with any sign show that electrostatic attraction is not the only effective factor, but other interactions that were mentioned are also effective in drug adsorption to particles.

### 3.3 Investigation of adsorption kinetics on XPDA carrier

Fig. 3(b) shows the kinetics of curcumin adsorption by XPDA. As depicted, the adsorption capacity initially exhibits a rapid increase, eventually reaching a state of equilibrium where the adsorption and release of curcumin are balanced. Subsequently, the adsorption rate slows down. These findings can be attributed to the availability of vacant sites for drug molecules in the initial stage, leading to a swift adsorption process. However, once a certain threshold is reached, steric hindrance becomes a factor, causing a decrease in the adsorption rate [[44], [45], [46], [47], [48]]. Furthermore, Fig. 3(c) also presents the investigation of equilibrium adsorption capacity for a concentration of 20 ppm at different pH levels. The measured value for pH ⁓2 was found to be 199.90 mg g^−1^.

According to Table 1 and the summary of experimental data, it is evident that the pseudo-second-order model is more suitable for investigating the adsorption kinetics compared to the pseudo-first-order model. This is supported by the higher correlation coefficient associated with the pseudo-second-order model. The calculated *q*_*e*_ values from the pseudo-second-order model align closely with the experimental *q*_*e*_ value, indicating that the pseudo-second-order model effectively describes the adsorption process. Based on the assumptions of the pseudo-second-order model, it can be inferred that the adsorption of curcumin molecules involves a chemical process, characterized by electron sharing between the carrier and the drug, as well as the formation of covalent forces on the adsorbent surface. Typically, the adsorption of a substance onto a solid surface can be governed by one or more steps, such as boundary layer infiltration, internal cavity infiltration, surface cavity infiltration, or a combination thereof, influencing the rate. When investigating the kinetics of penetration into the fixed particle, the parameter c represents the influence of the boundary layer [17,31,49]. Higher values of *c* indicate a greater impact of the boundary layer on the rate-controlling step. If the resulting graphs are linear and pass through the origin, interparticle diffusion solely controls the rate. However, in the case of XPDA, the non-linear graph suggests a two-stage adsorption process for curcumin (Fig. 3(c)). The initial region with a steeper slope corresponds to the penetration of curcumin molecules in the surface layer, while the subsequent region with a lower slope signifies the intraparticle penetration of drug molecules into the carrier cavities. Additionally, the fact that the straight lines of the graph do not intersect the origin indicates that while intraparticle diffusion significantly affects the adsorption rate, it is not the sole determining factor. Hence, both permeation in the surface layer and permeation within the particles contribute to the overall speed of the adsorption process [50,51]. According to Tables 1 and it is evident that the agreement in the first part of the diagram is more pronounced, indicating a lesser effect of the boundary layer on adsorption. Furthermore, the initial behavior of the intraparticle penetration model can be examined through the parameter *R*_*i*_, which represents the primary adsorption parameter of the intraparticle penetration model. The expression for *R*_*i*_ is shown in Equation (11):

(11) $$R_{i}=1-\left(\frac{c}{q_{ref}}\right)$$

**Table 1:** Adsorption kinetic parameters for XPDA.

Adsorption Kinetic	Parameters	XPDA
Pseudo-first Order	*K*_*1*_ (min^−1^)	0.033
	*q*_*e*_ (cal.) mg/g	51.240
	*q*_*e*_ (exp.) mg/g	199.900
	*R*^*2*^	0.919
Pseudo-second Order	*K*_*2*_ (g/mg.min)	0.002
	*q*_*e*_ (cal.) mg/g	192.300
	*q*_*e*_ (exp.) mg/g	199.900
	*R*^*2*^	0.999
Intraparticle penetration	*K*_*i1*_	0.007
	*C*_*1*_	156.749
	*R*^*2*^_*1*_	0.991
	*K*_*i2*_	0.0001
	*C*_*2*_	184.051
	*R*^*2*^_*2*_	0.088
	*R*_*i*_	0.079

In the above equation, *c* (mg/g) indicates intraparticle penetration and *q*_*ref*_ (mg/g) is the adsorption value at the reference time, which is assumed to be equal to the equilibrium adsorption capacity (*q*_*ref*_ *= q*_*e*_) in this study. Adsorption capacity can be analyzed with the help of *R*_*i*_ values based on four modes.

**Fig. 4:**
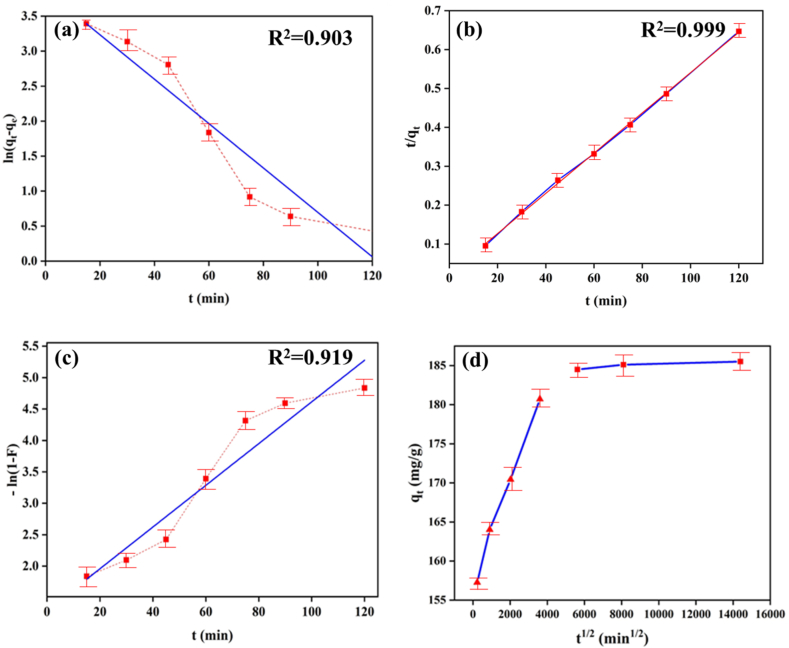
(a) Pseudo-first-order model, b) Pseudo-second-order model, c) Boyd model, d) Weber model.

1. 0.9<R_i_ < 1.0 related to weak initial adsorption;
2. 0.5< R_i_ < 0.9 average initial adsorption;
3. 0.1<R_i_ < 0.5 strong initial adsorption;
4. R_i_ < 0.1, almost full initial adsorption;

The value of *R*_*i*_ for XPDA is 0.08 which falls under state 4 and signifies the initial nearly complete adsorption. This observation can be attributed to the electrostatic interaction between the positive charge on the external surface and the negative charge of curcumin molecules. When examining Boyd's kinetic model, linear graphs that pass through the origin indicate that the rate of adsorption is governed by intraparticle penetration. Conversely, if the lines do not intersect the origin, it suggests that either penetration in the surface layer or a chemical reaction limits the adsorption rate. In Fig. 4, the lines do not cross the origin, indicating that interfacial penetration serves as the primary rate-controlling step in the drug adsorption process. This finding aligns with the results obtained from the pseudo-second-order model and intraparticle penetration analysis.

### 3.4 Adsorption isotherm

**Fig. 5:**
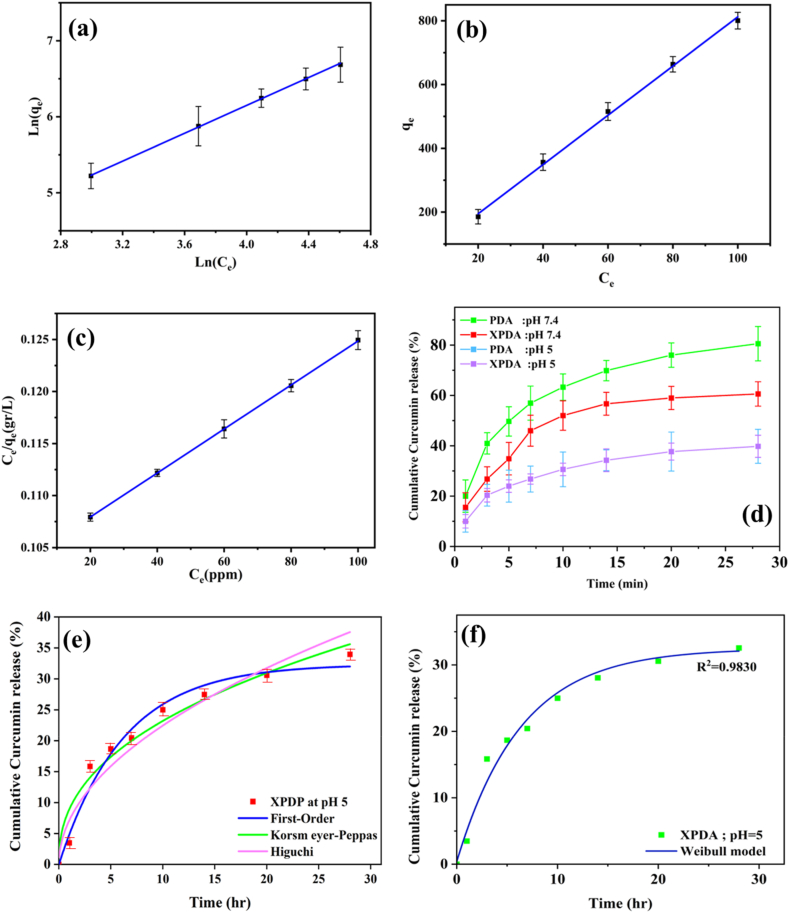
a) Adsorption isotherm, b) Freundlich model, c) Langmuir model for curcumin adsorption on XPDA. (d) Curcumin release kinetics from PDA and XPDA at different pH. (e, f) Models used to investigate release of curcumin from XPDA at pH 5.

Equilibrium adsorption isotherms play a crucial role in studying the surface properties of adsorbents and providing insights into the mechanisms of adsorption-surface processes. They offer valuable information regarding the characteristics of the adsorbent surface, adsorption behavior, design of adsorbent systems, and the relationship between the concentration of the adsorbed substance and the adsorbent's adsorption capacity. As depicted in Fig. 5(a–c), the adsorption capacity increases with the rise in the initial concentration of curcumin. This can be attributed to the greater concentration difference, which acts as a driving force in the adsorption process. In the Langmuir model, higher values of *K*_*L*_ indicate a strong bond between the drug and the carrier, while elevated *K*_*f*_ values suggest easy drug adsorption. The parameter *n/1* in the Freundlich model represents surface heterogeneity, and lower values of *n/1* indicate a more heterogeneous absorbent surface. Based on the values obtained from Table 2 regarding drug adsorption, the Langmuir model aligns closely with the experimental data. Furthermore, the correlation coefficient for the Langmuir model is higher than that of the Freundlich model, indicating a homogeneous adsorption process occurring on the surface, specifically the polydopamine layer. Notably, the values of *K*_*L*_ (l.mg^−1^) in Table 2, which amount to 493.33 l mg^−1^, signify a strong bond between the drug and XPDA. This can be attributed to the abundance of functional groups, including catechol and amine, present in the poly-dopamine layer, as well as electrostatic and π-π interactions that facilitate drug molecule adsorption. The fundamental principle of the Langmuir isotherm involves a dimensionless constant known as the separation factor (*R*_*L*_), which is defined by Equation (12):

(12) $$R_{L}=1/\left(1+bC_{0}\right)$$

In Equation (12), b is the constant related to the free energy of adsorption in terms of L.mg^−1^ and is obtained from the Langmuir model. According to R_L_, there are four types of adsorption processes.

1. If the separation factor value is greater than one, the adsorption process is unfavorable.
2. If the separation factor value is equal to one, the adsorption process is linear.
3. If the separation factor value is between zero and one, the adsorption process is favorable.
4. If the separation factor value is zero, the adsorption process is reversed.

**Table 2:** Isotherm parameters for curcumin adsorption.

Freundlich Isotherm Parameters			Langmuir Isotherm Parameters			Sample
R^2^	N	K_F_ (mg^1−(1/n)^ L^1/n^ g^−1^)	R^2^	K_L_ (L/mg)	q_m_ (mg/g)	
0.9995	1.0974	12.2154	0.9999	493.33	9.6525	XPDA

As can be seen in Table 2, for absorbents, the value of the separation factor is between zero and one, which indicates the desirability of the adsorption process.

### 3.5 Investigation of release properties

Fig. 5(d) presents kinetic diagrams illustrating the release of the curcumin drug from PDA and XPDA adsorbents at different pH levels. The graphs show an initial rapid release within the first hour, where approximately 60 % of the loaded curcumin is released at pH = 7.4. Afterward, the release rate decreases, resulting in a final release of around 80 % of the loaded molecule for PDA, while XPDA exhibits a more gradual and slower release. This observation indicates that the cross-links strengthen the structure of the polydopamine surface and enhance the stability of the system, leading to a controlled and gradual release of the loaded molecule [52,53]. To study the effect of environmental pH on the drug release profile, the release kinetics were examined at various pH levels. The graphs demonstrate that an increase in pH leads to a higher rate and amount of drug release. Over a duration of 28 h, the release increases from approximately 80 %–40 % for PDA, and from about 60 % to 35 % for XPDA as the pH transitions from 7.4 to 5. In both cases, the pH is above the isoelectric point, resulting in a negatively charged surface. The release occurs due to electrostatic repulsion, and with the increase in pH, the release becomes more pronounced. This can be attributed to a greater loss of protons from the amine groups in the polydopamine layer and the resulting negative-negative electrostatic repulsion between the curcumin anionic molecule and the polydopamine layer. As mentioned earlier, curcumin possesses anti-inflammatory effects and is suitable for tumor treatment. However, to exert beneficial effects on the body, it is necessary to minimize its toxicity and achieve a controlled and gradual release of the drug. Additionally, curcumin has very low solubility in water, leading to poor adsorption in the body. The presence of the polydopamine layer increases the hydrophilicity of curcumin, thereby enhancing its adsorption. To investigate the drug release behavior, the researchers utilized the first-order model, which is based on Equation (13):

(12) $$\frac{q_{t}}{q_{0}}=C_{0}-e^{-K_{F}t}$$

In this equation, q_t_/q_0_ represents the fraction of the doxorubicin drug released in the environment, K_F_ is the first-order rate constant, and C_0_ is the initial concentration of the drug. The release in this model depends on the gradient of drug concentration. Furthermore, the Korsmeyer-Peppas model was employed to determine the type of release mechanism. The model is represented by Equation (14):

(14) $$\frac{q_{t}}{q_{0}}=K_{R}t^{n}$$

In this equation, K_R_ is the release kinetic constant, and n is the release mechanism constant. The value of n determines the type of release mechanism. Specifically, if n equals 0.45, the release mechanism follows Fickian diffusion or the diffusion phenomenon. If n falls between 0.45 and 0.89, both diffusion and erosion mechanisms of the polymer matrix play a role in drug release. When n equals 0.89, drug release becomes time-independent, and in other cases, no specific mechanism can be attributed to drug release. If n is equal to 0.5, it is referred to as the Higuchi model, which assumes drug release is based on permeation, with K_H_ representing Higuchi's constant as depicted in Equation (15).

(15) $$\frac{q_{t}}{q_{0}}=K_{H}t^{1/2}$$

The obtained parameters for the aforementioned models are presented in Table 3. The value of n is determined 0.6227, indicating a mechanism involving penetration and gradual erosion of the matrix in drug release. The release rate is dependent on 0.377t, suggesting a non-Fickian type of drug transfer mechanism. Based on these results, the drug release from XPDA adheres to both the Higuchi and first-order models, indicating that the primary mechanism for drug release is permeation. Fig. 5(e) shows the models used to investigate curcumin release from XPDA. For the Weibull model shown in Fig. 5(f), the excellent fit (R^2^ = 0.9830) suggests this model best describes the release kinetics, particularly capturing both the initial burst release and the plateau phase. The Weibull model's superior fit indicates that the drug release mechanism likely involves a complex combination of diffusion, erosion, and matrix relaxation processes [54,55].

**Table 3:** Kinetic parameters of drug release.

	First-order model	Korsmeyer-Peppas model	Higuchi model
**R**^**2**^**correlation coefficient**	0.9782	0.7547	0.9461
**The coefficients of equation**	K_F_ = 0.1608	K_R_ = 5.4162	K_H_ = 7.1021
	C_0_ = 32.36	n = 0.6227	

### 3.6 Model energy optimization

In this study, the modified macromolecular structure model of materials was imported into Materials Studio 2020 software. The simulation employed the COMPASS force field to evaluate the convergence and energy states of the polymer under investigation, with the objective of attaining its utmost stability. Convergence is a method or algorithm employed to investigate molecular systems, referring to the point at which the simulation reaches a stable result that remains consistent under repeated calculations or further adjustments. Additionally, the energy of the system is minimized during the calculations by adjusting the atomic positions based on the forces and differences obtained from the quantum calculations. Table 4 shows the solubility parameters (${\left(\frac{J}{cm^{3}}\right)}^{0.5}$) derived from Material Studio for three mentioned materials. This information was then used for evaluating drug loading performance.

**Table 4:** Solubility parameters.

Material	Solubility Parameter	Standard Error
Dopamine	12.488	0.177
Curcumin	18.321	0.839

**Fig. 6:**
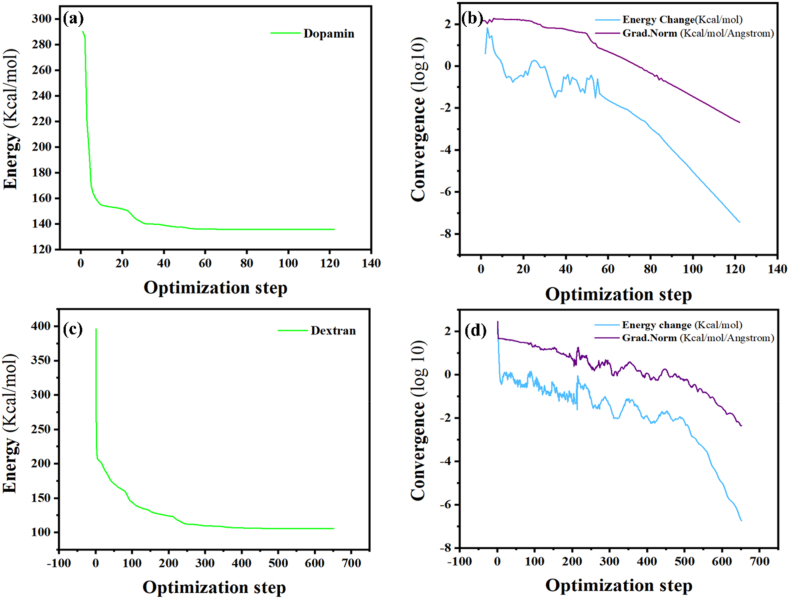
(a, b) Forcite geometry optimizations for the repeating unit of Dopamine. (c, d) Forcite geometry optimizations for the repeating unit of Dextran.

The calculations involved synthesized polydopamine and dextran, each made up of three repeating units. Fig. 6(a) and Fig. 6(c) show a consistent decrease in energy levels until the most stable state is reached. In addition, Fig. 6(b) and (d) illustrate the examination of all possible conditions, ultimately leading to the system's most stable configuration.

**Fig. 7:**
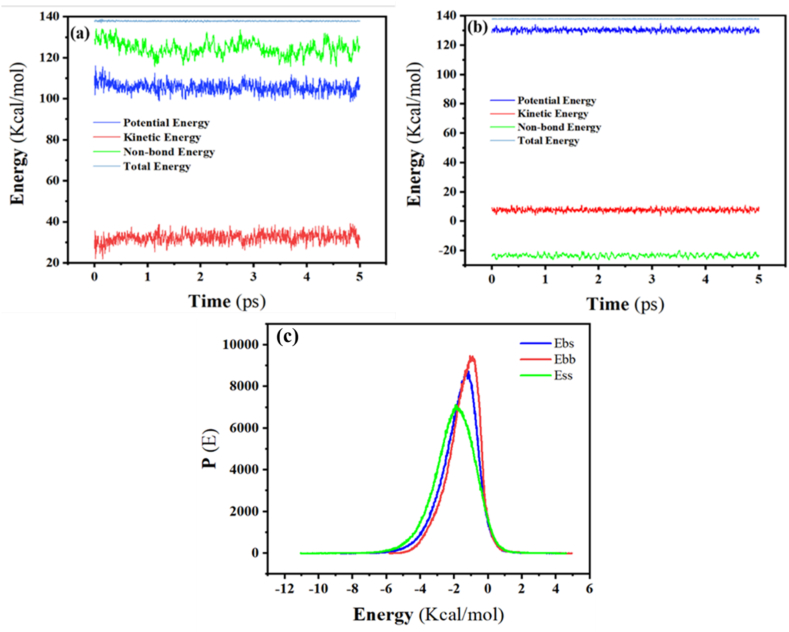
(a) Forcite dynamic energies for three repeating units of poly dextran (b) Forcite dynamic energies for the repeating unit of Dopamine (c) Blend Binding Energy Distribution Curve.

There are various methods available to determine equilibrium state. One of these methods involves controlling and monitoring the time evolution of system characteristics, such as temperature and energy. During equilibrium, these characteristics exhibit fluctuations around their average values while remaining relatively constant over time. Equilibrium is achieved when these characteristics stabilize and reach a constant value. For this reason, dynamic tests were conducted using two modes: constant temperature (NVT) and constant energy (NVE) as depicted in Fig. 7(a) and (b). These tests aim to enhance the stability of the molecule and prepare it for subsequent processes by facilitating the creation of dynamic energy. In the NVT test, a constant temperature was simulated during the interaction, enabling the study of temperature-dependent properties. On the other hand, the NVE test simulates the energy of the entire system during the interaction, ensuring that the system is isolated and does not exchange energy with any external sources, thereby maintaining a constant total energy.

In the subsequent steps, after preparing the molecule, the forcite module was utilized to calculate the adhesion energy density and the solubility parameter, with the corresponding information presented in Table 5, Table 6. Using this module and the provided information, it becomes possible to obtain the energy of the entire system and its intramolecular energies.

**Table 5:** Cohesive energy density and solubility for the repeating unit of Dopamine.

	Cohesive energy density (J/m^3^)	Standard error (J/m^3^)	Solubility parameter (J/cm^3^)^0.5^	Standard error (J/cm^3^)^0.5^	Computing intermolecular energies
**Total**	5.306e+008	1.505e+007	23.032	0.327	−14.962
**Van der Waals**	3.535e+008	1.299e+005	18.802	0.003	−9.690
**Electrostatic**	1.656e+008	1.518e+007	12.854	0.591	−4.956
**Other**	1.150e+007	0.000e+000	3.391	0.000	–

**Table 6:** Cohesive energy density and solubility for three repeating units of poly dextran.

	Cohesive energy density (J/m^3^)	Standard error (J/m^3^)	Solubility parameter (J/cm^3^)^0.5^	Standard error (J/cm^3^)^0.5^	Computing intermolecular energies
**Total**	4.225e+008	5.234e+007	20.516	1.275	−39.287
**Van der waals**	1.816e+008	5.033e+006	13.475	1.275	−18.740
**Electrostatic**	2.344e+008	4.730e+007	15.231	1.553	−19.855
**Other**	6.523e+006	0.000e+000	2.554	0.0	–

In Fig. 7(c), the blend binding energy distribution curve shows the distribution of binding energy in a mixture or combination of molecules. It provides information about the range of binding energies and the relative percentages of each binding with a specific energy in a compound for polydopamine (base) and dextran (screen). The closeness of the blend binding energy distribution curves for different blends offers insights into the similarity or dissimilarity of binding interactions. The resulting curves show significant overlap, indicating the similarity between the binding energy and the interaction energy of the molecules. The comparable distribution of these curves at 298 K demonstrates the compatibility of these structures with each other. This shows that the parameters of these materials are closely aligned, making them suitable for being placed next to each other. Consequently, the polymer is capable of effectively loading the drug.

**Fig. 8:**
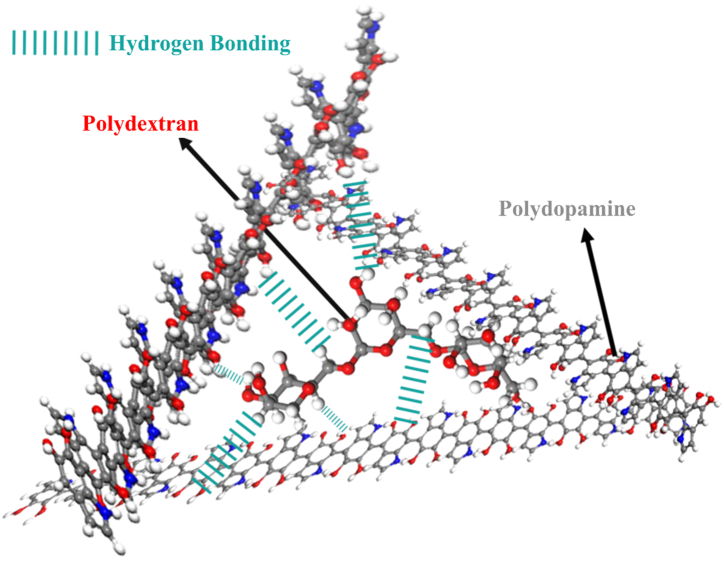
A 3D image containing the structures currently being sampled.

Furthermore, the Chi parameter (*ꭓ*) has been determined by examining various states, including chain length, for both the dextran monomer and dextran with 3 repeating units, as well as the synthesized polydopamine. The obtained results reveal that the chi (*ꭓ*) value ranges from −0.142 to −0.900, with the best result being −0.900 for the dextran with 3 units and polydopamine. Fig. 8 shows a 3D image containing polydextran and polydopamine.

## 4 Conclusions

In this study, cross-linked polydopamine particles (XPDPs) were synthesized using a water-in-water Pickering emulsion system, demonstrating their potential as an effective drug delivery carrier for curcumin. The cross-linking process, which employed EDC and PAA, enhanced the structural integrity and stability of the polydopamine particles, leading to improved drug loading efficiency and a more controlled release profile compared to non-crosslinked PDA particles. The results showed that the pH-sensitive nature of XPDPs allowed for a gradual release of curcumin, with the release rate increasing in higher pH environments due to electrostatic repulsion. Molecular simulations confirmed the compatibility between PDA and dextran, supporting the high adsorption capacity of XPDPs. Additionally, kinetic studies revealed that curcumin adsorption followed a pseudo-second-order model, indicating that chemical adsorption involving electron sharing played a key role. The Langmuir isotherm model, with its high correlation coefficient, further confirmed that the adsorption process occurred on a homogeneous surface with strong drug-carrier interactions. Overall, XPDPs exhibit promising characteristics for sustained drug delivery applications, offering improved structural stability and controlled drug release, which can be fine-tuned by manipulating environmental factors such as pH. These findings highlight the potential of XPDPs in biomedical applications, particularly for targeted therapies requiring precise control over drug release rates. Further studies exploring other drug molecules and in vivo applications could expand the utility of XPDPs in clinical settings.

## CRediT authorship contribution statement

**Majid Moussaei:** Investigation. **Ebrahim Tajik:** Investigation. **Vahid Haddadi-Asl:** Writing – review & editing, Validation. **S. Ali Mazloumi:** Resources. **Helia Heydarinasab:** Data curation. **Elahe Abdollahi:** Resources. **Fatemeh Haj-Sadeghi:** Resources. **Hanie Ahmadi:** Writing – original draft. **Mohammad Reza Gholizadeh:** Validation.

## Data availability

Data will be made available on request.

## Funding

This research received no grant.

## Declaration of competing interest

The authors declare that they have no known competing financial interests or personal relationships that could have appeared to influence the work reported in this paper.

## References

[1] Acter S., Vidallon M.L.P., Crawford S., Tabor R.F., Teo B.M. (2021). Bowl-shaped mesoporous polydopamine nanoparticles for size-dependent endocytosis into HeLa cells. ACS Appl. Nano Mater..

[2] Dashtdar A., Yazadani-Ahmadabadi H., Rezvani-Moghaddam A., Salami-Kalajahi M., Sundararaj U. (2024). Scalable polydopamine coatings with increased thickness and stability using polyamidoamine dendrimers. Appl. Surf. Sci..

[3] Lin K., Gan Y., Zhu P., Li S., Lin C., Yu S., Zhao S., Shi J., Li R., Yuan J. (2021). Hollow mesoporous polydopamine nanospheres: synthesis, biocompatibility and drug delivery. Nanotechnology.

[4] Liu Y., Zhang Y., Wang J., Yang H., Zhou J., Zhao W. (2022). Doxorubicin-loaded walnut-shaped polydopamine nanomotor for photothermal-chemotherapy of cancer. Bioconjug. Chem..

[5] Khezraqa H., Safavi-Mirmahalleh S.-A., Roghani-Mamaqani H., Salami-Kalajahi M. (2024). A review on polydopamine as an efficient material in different components of rechargeable ion batteries. J. Energy Storage.

[6] Yan S., Huang Q., Chen J., Song X., Chen Z., Huang M., Xu P., Zhang J. (2019). Tumor-targeting photodynamic therapy based on folate-modified polydopamine nanoparticles. Int. J. Nanomedicine.

[7] Xu H., Zhang Y., Zhang H., Zhang Y., Xu Q., Lu J., Feng S., Luo X., Wang S., Zhao Q. (2023). Smart polydopamine-based nanoplatforms for biomedical applications: state-of-art and further perspectives. Coord. Chem. Rev..

[8] Liu Y., Ai K., Lu L. (2014). Polydopamine and its derivative materials: synthesis and promising applications in energy, environmental, and biomedical fields. Chem. Rev..

[9] Lazar S., Shen R., Quan Y., Palen B., Wang Q., Ellison C.J., Grunlan J.C. (2021). Mixed solvent synthesis of polydopamine nanospheres for sustainable multilayer flame retardant nanocoating. Polym. Chem..

[10] Bolat G., Vural O.A., Yaman Y.T., Abaci S. (2021). Polydopamine nanoparticles-assisted impedimetric sensor towards label-free lung cancer cell detection. Mater. Sci. Eng. C.

[11] Liebscher J., Mrówczyński R., Scheidt H.A., Filip C., Hădade N.D., Turcu R., Bende A., Beck S. (2013). Structure of polydopamine: a never-ending story?. Langmuir.

[12] Uthappa U.T., Kigga M., Sriram G., Ajeya K.V., Jung H.-Y., Neelgund G.M., Kurkuri M.D. (2019). Facile green synthetic approach of bio inspired polydopamine coated diatoms as a drug vehicle for controlled drug release and active catalyst for dye degradation. Microporous Mesoporous Mater..

[13] Cheng H., He Y., Lu J., Yan Z., Song L., Mao Y., Di D., Gao Y., Zhao Q., Wang S. (2023). Degradable iron-rich mesoporous dopamine as a dual-glutathione depletion nanoplatform for photothermal-enhanced ferroptosis and chemodynamic therapy. J. Colloid Interface Sci..

[14] Yegappan R., Selvaprithiviraj V., Mohandas A., Jayakumar R. (2019). Nano polydopamine crosslinked thiol-functionalized hyaluronic acid hydrogel for angiogenic drug delivery. Colloids Surf. B Biointerfaces.

[15] Ko E., Lee J.S., Kim H., Yang S.Y., Yang D., Yang K., Lee J., Shin J., Yang H.S., Ryu W., Cho S.-W. (2018). Electrospun silk fibroin nanofibrous scaffolds with two-stage hydroxyapatite functionalization for enhancing the osteogenic differentiation of human adipose-derived mesenchymal stem cells. ACS Appl. Mater. Interfaces.

[16] Tsai W.-B., Chen W.-T., Chien H.-W., Kuo W.-H., Wang M.-J. (2011). Poly(dopamine) coating of scaffolds for articular cartilage tissue engineering. Acta Biomater..

[17] Roshan Z., Haddadi‐Asl V., Ahmadi H., Moussaei M. (2024). Curcumin‐encapsulated poly(lactic‐ *co* ‐glycolic acid) nanoparticles: a comparison of drug release kinetics from particles prepared via electrospray and nanoprecipitation. Macromol. Mater. Eng..

[18] Fallahi-Samberan M., Salami-Kalajahi M., Dehghani E., Abbasi F. (2019). Investigating Janus morphology development of poly(acrylic acid)/poly(2-(dimethylamino)ethyl methacrylate) composite particles: an experimental study and mathematical modeling of DOX release. Microchem. J..

[19] Jia S., Tang D., Zhou Y., Du Y., Peng J., Sun Z., Yang X. (2020). Polydopamine microsphere-incorporated electrospun fibers as novel adsorbents for dual-responsive adsorption of methylene blue. ACS Appl. Mater. Interfaces.

[20] Rostamipoor H., Ahmadi H., Haddadi-Asl V., Moussaei M., Roghani-Mamaqani H. (2024). Synthesis of magnetic polystyrene nanocomposite by emulsion polymerization using a photo-responsive emulsifier for efficient crude oil absorption. J. Photochem. Photobiol. Chem..

[21] Ahmadi H., Moussaei M., Haddadi-Asl V., Roghani-Mamaqani H. (2024). Synthesis of multi-stimuli-responsive flocculants using reversible addition-fragmentation chain transfer polymerization: investigating the performance and mechanism of kaolin coagulation. Polymer.

[22] Wang Y., Ge W., Ma Z., Ji G., Wang M., Zhou G., Wang X. (2022). Use of mesoporous polydopamine nanoparticles as a stable drug-release system alleviates inflammation in knee osteoarthritis. APL Bioeng..

[23] Li Z., Li S., Yu D., Zhang F. (2024). Elucidating pH dynamics in Pickering emulsions stabilized by soybean protein isolate and nicotinamide mononucleotide: enhancing emulsification and curcumin delivery mechanisms. Colloids Surf. A Physicochem. Eng. Asp..

[24] Li Z., Zhao R., Hu F., Zhang Y., Dong B., Lu P., Song Z., Wang H., Zhang F., Liu W., Yu D., Li H. (2024). Pickering emulsions stabilized by whey protein isolate/nicotinamide mononucleotide complex and their application in pH-responsive drug delivery. Ind. Crops Prod..

[25] Ding H., Tan P., Fu S., Tian X., Zhang H., Ma X., Gu Z., Luo K. (2022). Preparation and application of pH-responsive drug delivery systems. J. Controlled Release.

[26] Nishizawa N., Kawamura A., Kohri M., Nakamura Y., Fujii S. (2016). Polydopamine particle as a particulate emulsifier. Polymers.

[27] Li Z., Yu D. (2023). Controlled ibuprofen release from Pickering emulsions stabilized by pH-responsive cellulose-based nanofibrils. Int. J. Biol. Macromol..

[28] Dehghani B., Hosseini M.S., Salami-Kalajahi M. (2020). Neutral pH monosaccharide receptor based on boronic acid decorated poly(2-hydroxyethyl methacrylate): spectral Methods for determination of glucose-binding and ionization constants. Microchem. J..

[29] Fallahi-Sambaran M., Salami-Kalajahi M., Dehghani E., Abbasi F. (2018). Investigation of different core-shell toward Janus morphologies by variation of surfactant and feeding composition: a study on the kinetics of DOX release. Colloids Surf. B Biointerfaces.

[30] Torkpur-Biglarianzadeh M., Salami-Kalajahi M. (2015). Multilayer fluorescent magnetic nanoparticles with dual thermoresponsive and pH-sensitive polymeric nanolayers as anti-cancer drug carriers. RSC Adv..

[31] Abdollahi E., Khalafi-Nezhad A., Mohammadi A., Abdouss M., Salami-Kalajahi M. (2018). Synthesis of new molecularly imprinted polymer via reversible addition fragmentation transfer polymerization as a drug delivery system. Polymer.

[32] Mohammadzadeh F., Golshan M., Haddadi-Asl V., Salami-Kalajahi M. (2023). Adsorption kinetics of methylene blue from wastewater using pH-sensitive starch-based hydrogels. Sci. Rep..

[33] Mohammadzadeh F., Haddadi-Asl V., Salami-Kalajahi M. (2024). pH-sensitive multi-arm star polyampholytes: a novel approach for simultaneous adsorption of anionic and cationic dyes. J. Mol. Liq..

[34] Mohammadzadeh F., Haddadi-Asl V., Salami-Kalajahi M. (2024). Block-copolymer-armed star polyampholyte with pH- and temperature-tunable supramolecular nanostructures for enhanced dye adsorption. Colloids Surf. A-Physicochem. Eng. Asp..

[35] Alizadeh M., Abdi S., Abdoli S.M., Hazrati H., Salami-Kalajahi M. (2024). Investigating the adsorption of humic acid from water using CTS/PAM and CTS/PAM/EDTA adsorbents. Int. J. Chem. Eng..

[36] Tsakos M., Schaffert E.S., Clement L.L., Villadsen N.L., Poulsen T.B. (2015). Ester coupling reactions – an enduring challenge in the chemical synthesis of bioactive natural products. Nat. Prod. Rep..

[37] Gholizadeh M.R., Haddadi-Asl V., Ahmadi H., Moussaei M. (2024). Organic dyes and ions selective adsorption and separation from aqueous solution using novel synthetic cross-linked-polydopamine/polyaniline nanoparticles. Chem. Pap..

[38] Nikdel M., Salami-Kalajahi M., Hosseini M.S. (2014). Synthesis of poly(2-hydroxyethyl methacrylate-co-acrylic acid)-grafted graphene oxide nanosheets via reversible addition-fragmentation chain transfer polymerizations. RSC Adv..

[39] Panahian P., Salami-Kalajahi M., Hosseini M.S. (2014). Synthesis of dual thermoresponsive and pH-sensitive hollow nanospheres by atom transfer radical polymerization. J. Polym. Res..

[40] Lakouraj M.M., Rezaei M., Hasantabar V. (2021). Synthesis, characterization and in-vitro prolonged release of L-DOPA using a novel amphiphilic hydrogel based on sodium alginate-polypyrrole. Int. J. Biol. Macromol..

[41] Zeynalzadeh S., Dehghani E., Hassani A., Khoshfetrat A.B., Salami-Kalajahi M. (2024). Effect of curcumin-loaded poly(amidoamine) dendrimer on cancer cell lines: a comparison between physical loading and chemical conjugation of drug. Polym. Bull..

[42] Dehghani E., Barzgari-Mazgar T., Salami-Kalajahi M., Kahaie-Khosrowshahi A. (2020). A pH-controlled approach to fabricate electrolyte/non-electrolyte janus particles with low cytotoxicity as carriers of DOX. Mater. Chem. Phys..

[43] Panahian P., Salami-Kalajahi M., Hosseini M.S. (2014). Synthesis of dual thermosensitive and pH-sensitive hollow nanospheres based on poly(acrylic acid-b-2-hydroxyethyl methacrylate) via an atom transfer reversible addition-fragmentation radical process. Ind. Eng. Chem. Res..

[44] Song H., Tian K., Fang Z., Guan C., Jiang H., Lu M., Zhang M., Zhuang S., Wei H., Wei D., Li X. (2023). Steric-hindrance effect and self-sacrificing template behavior induced PDA@SnO2-QDs/N-doped carbon hollow nanospheres: enhanced structural stability and reaction kinetics for long-cyclic Li-ion half/full batteries. J. Colloid Interface Sci..

[45] Xu C., Shan Y., Bilal M., Xu B., Cao L., Huang Q. (2020). Copper ions chelated mesoporous silica nanoparticles via dopamine chemistry for controlled pesticide release regulated by coordination bonding. Chem. Eng. J..

[46] Golshan M., Akbari-Meinagh M., Alizadeh A.A., Salami-Kalajahi M. (2024). Synthesis and self-assembly of perylene-cored poly(amidoamine) dendrimers with poly[2-(dimethylamino)ethyl methacrylate]-modified arms as fluorescent bio-imaging probes and doxorubicin carriers. Mater. Chem. Phys..

[47] Liu Z., Wu Y., Lan F., Xie G., Zhang M., Ma C., Jia J. (2023). Improvement of permeability and antifouling performance of PVDF membranes via dopamine-assisted deposition of zwitterionic copolymer. Colloids Surf. A Physicochem. Eng. Asp..

[48] Ghaffari S., Golshan M., Jalili K., Salami-Kalajahi M. (2024). Anti-inflammatory drugs-modified poly(2-hydroxyethyl methacrylate) particles as anticancer drug carriers. Macromol. Mater. Eng..

[49] Valverde A., Cabrera-Codony A., Calvo-Schwarzwalder M., Myers T.G. (2024). Investigating the impact of adsorbent particle size on column adsorption kinetics through a mathematical model. Int. J. Heat Mass Transf..

[50] Abdollahi E., Haddadi‐Asl V., Ahmadi H., Shirjandi M., Khanipour F. (2024). Investigation of release kinetics of DOX from polydopamine nanocapsules prepared by hard template method. Macromol. Mater. Eng..

[51] Nikravan G., Haddadi-Asl V., Salami-Kalajahi M. (2018). Synthesis of dual temperature– and pH-responsive yolk-shell nanoparticles by conventional etching and new deswelling approaches: DOX release behavior. Colloids Surf. B Biointerfaces.

[52] Yang M., Lee S.Y., Kim S., Koo J.S., Seo J.-H., Jeong D.I., Hwang C., Lee J., Cho H.-J. (2020). Selenium and dopamine-crosslinked hyaluronic acid hydrogel for chemophotothermal cancer therapy. J. Controlled Release.

[53] Guo T., Wang W., Song J., Jin Y., Xiao H. (2021). Dual-responsive carboxymethyl cellulose/dopamine/cystamine hydrogels driven by dynamic metal-ligand and redox linkages for controllable release of agrochemical. Carbohydr. Polym..

[54] Dong M., Liu W., Yang Y., Xie M., Yuan H., Ni C. (2023). Load and release of gambogic acid via dual-target ellipsoidal-Fe_3_ O_4_ @SiO_2_ @mSiO_2_ -C_18_ @dopamine hydrochloride -graphene quantum dots-folic acid and its inhibition to VX2 tumor cells. Nanotechnology.

[55] Septian Dwitya S., Lin K.-S., Weng M.-T., Vukile Mdlovu N., Tsai W.-C., Wu C.-M. (2024). Synthesis and characterization of pH-triggered doxorubicin-conjugated polydopamine-coated cobalt ferrite nanoparticles for in-vitro/in-vivo studies in liver cancer therapy. J. Ind. Eng. Chem..

