# Supplementary material for: Achieving enhanced stabilization and controlled release of curcumin via cross-linked polydopamine particles
Source: Heliyon. 2024 Dec 20;11(1):e41379. doi: 10.1016/j.heliyon.2024.e41379 (PMC11729636; doi:10.1016/j.heliyon.2024.e41379)
Supplement: Multimedia component 1 [file mmc1.docx]

**Supplementary Materials**

**Achieving Enhanced Stabilization and Controlled Release of Curcumin *via* Cross-Linked Polydopamine Particles**

Majid Moussaei^1^, Ebrahim Tajik^1^, Vahid Haddadi-Asl^*1^, S. Ali Mazloumi^1^, Helia Heydarinasab^1^, Elahe Abdollahi^1^, Fatemeh Haj-Sadeghi^1^, Hanie Ahmadi^1^, Mohammad Reza Gholizadeh^1^

^1^Department of Polymer Engineering and Color Technology, Amirkabir University of Technology, P.O. Box 15875-4413, Tehran, Iran.

Corresponding author: [haddadi@aut.ac.ir](mailto:haddadi@aut.ac.ir)

The XRD spectra of PDA is presented in **Figure S1**. As can be seen, there is a broad peak in 2𝜃 ≈20˚-30˚. This peak is related to the amorphous or semi-crystalline structure of polydopamine, which is observed due to the random arrangement of its molecules. The width of this peak confirms the broadness of the crystalline order and indicates that the structure is mostly amorphous [1,2]


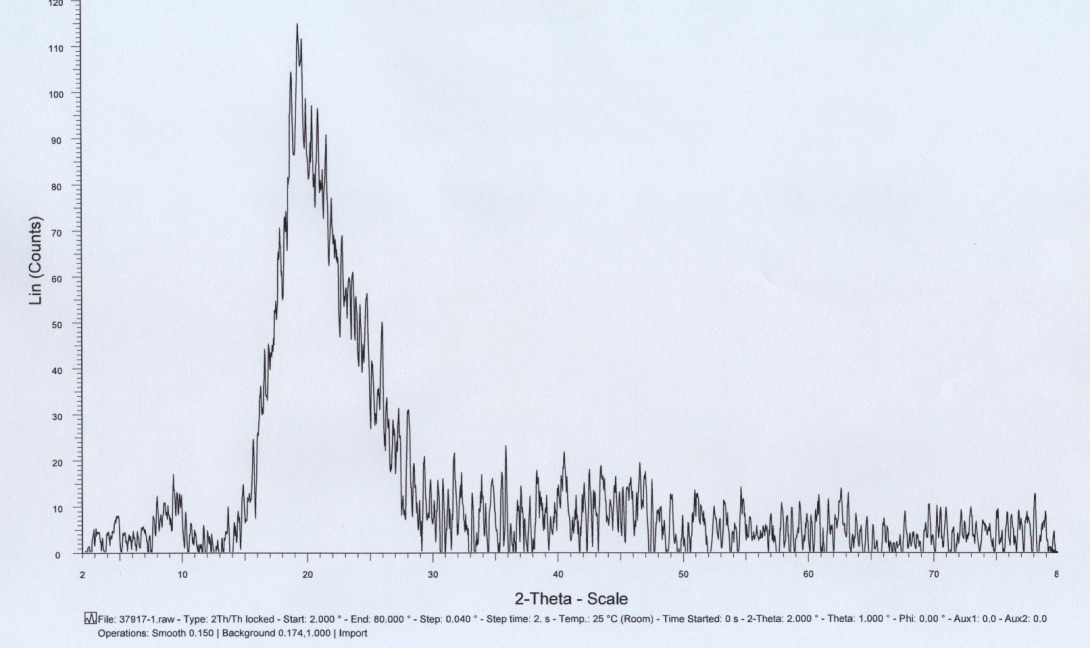


**Figure S1. XRD data for PDA**

**Figure S2** shows the zeta potential values for XPDPs at various pH. As can be seen the zeta potential is 8.9 mV at pH about 2 , **Figure S2(a)**. With the increase in pH, the value of zeta potential decreased to about 0.5 mV and finally reached to -1.9mV in alkaline environment, **Figure S2(b,c)**. But the values are not very high, which shows that the mechanism governing the adsorption mechanism are van der Waals interactions and π-π interactions, in addition to electrostatic forces.


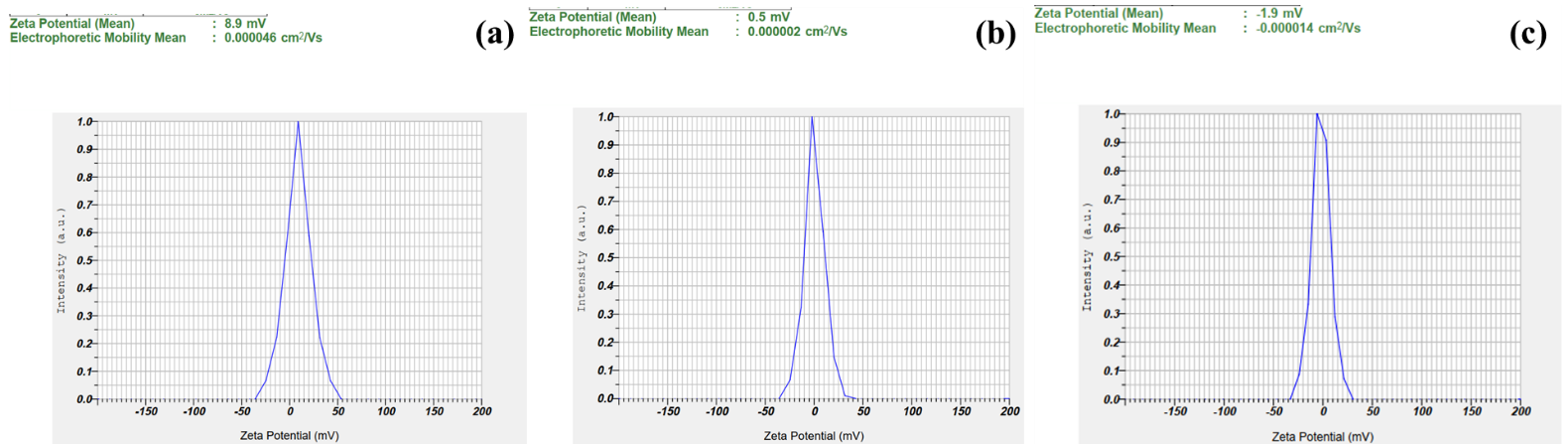


**Figure S2. Zeta Potential measurement for XPDPs at different pH values.**

**References**

[1] J.C. García-Mayorga, H.-C. Rosu, A.B. Jasso-Salcedo, V.A. Escobar-Barrios, Kinetic study of polydopamine sphere synthesis using TRIS: relationship between synthesis conditions and final properties, RSC Adv. 13 (2023) 5081–5095. https://doi.org/10.1039/D2RA06669F.

[2] X. Han, X. Chen, M. Yan, H. Liu, Synergetic effect of polydopamine particles and in-situ fabricated gold nanoparticles on charge-dependent catalytic behaviors, Particuology 44 (2019) 63–70. https://doi.org/10.1016/j.partic.2018.07.004.
